# Modelling the effect of moose *Alces alces* population density and regional forest structure on the amount of damage in forest seedling stands

**DOI:** 10.1002/ps.6081

**Published:** 2020-09-28

**Authors:** Ari Nikula, Juho Matala, Ville Hallikainen, Jyrki Pusenius, Antti Ihalainen, Tuomas Kukko, Kari T Korhonen

**Affiliations:** ^1^ Natural Resources Institute Finland Rovaniemi Finland; ^2^ Natural Resources Institute Finland Joensuu Finland; ^3^ Natural Resources Institute Finland Helsinki Finland; ^4^ Natural Resources Institute Finland Jyväskylä Finland

**Keywords:** *Alces alces*, forestry, moose, moose damage risk, population density

## Abstract

**BACKGROUND:**

Moose (*Alces alces* L.) populations and moose damage in forests are debated in Nordic countries with dense moose populations. Moose populations and food resources vary greatly, both spatially and temporally, and reliable data covering both variables simultaneously at the same scale have seldom been available. We modelled the effect of moose population density and forest resources on the area of moose damage at regional scale, referring to moose management areas (MMA). Forest data and moose damage data originated from the Finnish National Forest Inventory, and the moose population data came from a Bayesian moose model. For modelling, average values of moose population, damage and forest variables were calculated for the periods 2004–2008 and 2009–2013 for each MMA. The MMAs were further classified into one of four larger geographical zones. The area of moose damage was used as a dependent variable, and the proportions of different types of forests and moose population densities per land area or area of seedling stands as explanatory variables. The relationships were modelled with a linear mixed‐effects model with an exponential spatial correlation structure.

**RESULTS:**

The area of moose damage was best explained by total forest area, proportions of plantations and mature forests, and moose population density per land area or the proportion of plantations. There were differences among the biogeographical zones in how different variables explained the amount of damage.

**CONCLUSION:**

The results provide tools for analyzing the regional effects of moose population density and the amount of food resources on the amount of moose damage. This information can be used in reconciling sustainable moose population levels and the amount of damage.

## Graphical abstract

We modelled the amount of moose damage with linear mixed effects models by using regional forest structure parameters. The model can be used as a tool in moose management.

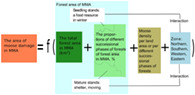

© 2020 The Authors. *Pest Management Science* published by John Wiley & Sons Ltd on behalf of Society of Chemical Industry.

## 1 INTRODUCTION

Ungulate species have increased throughout Europe during the last few decades and become locally overabundant.^1^ Ungulates provide benefits for humans in terms of hunting and recreational value, but locally, high numbers of ungulates also cause considerable damage to forestry and agriculture, and cause thousands of ungulate–vehicle collisions.^1^, ^2^ The largest ungulate species, moose (*Alces alces* L.) has an ambiguous position in Fennoscandian (Sweden, Norway and Finland) nature because it is the most important game animal, but it also causes considerable losses to forest owners.^3^, ^4^ Taking the large amount of moose damage and consequent economic losses in forests into account, the term ‘pest’ also can be applied to moose.^5^ To reconcile the benefits and costs of ungulates, there is a need for tools that can assess the impact of different population levels on benefits and costs at scales feasible for ungulate management.^1^, ^2^

Rapid growth of moose populations occurred in all Fennoscandian countries at the beginning of the 1970s.^6^, ^7^, ^8^ The winter population in Finland was ≈30 000 moose at the beginning of 1970 and increased to ≈120 000 in the early 1980s. At the same time, the moose populations were ≈300 000 in Sweden and ≈90 000 in Norway, and these have remained about the same up to the present.^6^, ^8^ After the 1980s, the moose population decreased in Finland until the mid‐1990s and peaked again at the turn of the millennium, when it was ≈160 000. Since then, the overwintering population has been ≈90 000 moose.

The most intensive growth in moose populations occurred a couple of decades after a modern forest management system with clear‐cutting and planting of mainly coniferous trees was applied in Nordic forestry at the beginning of the 1950s. The applied forestry methods have resulted in a continuously large proportion of young development classes of coniferous trees, especially Scots pine (*Pinus sylvestris* L.) dominated forests, which has benefitted moose in terms of suitable winter foraging areas.^6^, ^9^

The increased moose populations have subsequently caused increasing damage to forests.^4^, ^10^, ^11^ In Finland, moose damage was recorded on 960 000 ha of forest land in the 11th National Forest Inventory (NFI) in 2009–2013.^11^ In this area, the quality of plantation decreased in 520 000 ha and serious damage was recorded in 106 000 ha. The majority (75%) of damage occurred in Scots pine‐dominated stands, among which serious damage covered 85 000 ha, corresponding to ≈22% of Scots pine plantations. However, the highest proportion of damaged stands were found in birch species (*Betula pendula* Roth. and *Betula pubescens* Ehrh.) and other stands dominated by broadleaved species.^11^ In Sweden, moose damage was found in 12–15% of Scots pine plantations in 2003–2013.^12^ Also in Norway, moose and other ungulates have been among the most severe damage agents, and moose have been estimated to cause annual losses of EUR 1.5–3.7 million.^2^, ^13^

A plethora of factors, such as human disturbance, snow depth, topography and soil, forest landscape composition, tree species composition and spatial structure of plantations, and competition from other deer species among others, have been shown to affect moose browsing and habitat selection at the habitat and home‐range levels.^14^, ^15^, ^16^, ^17^, ^18^, ^19^ Moose are facultative food specialists/generalists, because the diet of moose consists of woody species, mainly Scots pine, in winter, but in summer, moose utilize tens of species of plants and also browse in seedling stands.^20^, ^21^, ^22^ Although deciduous trees are preferred and Scots pine is only of medium preference, pine forms the majority of moose diet in winter, and consequently, most of the damage occurs to pine.^11^, ^23^, ^24^

Moose cause damage to trees by browsing leader and lateral shoots, breaking stems and stripping bark.^25^ As a consequence, the growth of trees is reduced and the quality of timber is impaired.^26^ In the worst cases, the whole seedling stand has to be regenerated. All types of damage cause considerable economic losses to forest owners as a consequence of the lower amount of timber during the rotation period and lower prices of timber owing to impaired quality.^26^, ^27^

Different types of repellents have been tested for controlling moose damage, but the costs and the amount of labour are considerable because control has to continue for several years until the seedlings grow beyond the reach of moose.^28^, ^29^ Mechanical protection methods, such as fencing, has been tested, but although rather effective, their costs are high, and they are thus applicable only in special cases.^30^ In summary, owing to the high amount of seedling stands prone to damage, the most feasible method of controlling the amount of damage at regional and country level is the management of moose populations.

Key information in managing wildlife populations for keeping damage at a sustainable and acceptable level is the effect of different population densities on the amount of damage. However, only a few studies have been able to assess the correlation between moose population and damage at scales applicable to moose management.^31^ Apparently, the relationship between moose population and damage has been a problematic subject for study owing to the lack of data on moose populations, moose damage and food resources at the same spatial and temporal scales. The results indicate that the amount of moose damage at national and regional levels reflects changes in moose population levels.^31^ In addition, the changes in moose populations also are reflected in the changes of browsing on preferred tree species and damage in pine stands.^31^, ^32^ The moose density index has been found to positively correlate with browsing pressure on Scots pine also at local scales, but neither evidence of density‐dependent habitat selection nor correlation between population level and damage level has been found.^31^, ^33^ Rather, significant correlation between the availability of browse species and the browsing intensity of the most preferred food have been found at regional and at landscape levels similar to, or larger than, the home range sizes of moose.^34^, ^35^

The aim of this work was to model how the variation in moose population and forest resources explain browsing damage in forest plantations at scales used in moose management – moose management areas (MMAs). We hypothesized that the amount of moose damage is dependent on population density in relation to the amount and composition of forests dominated by different tree species, representing young successional stages of forests, mature forests and forestry land. Furthermore, we hypothesized that the effect of these factors explain damage in different ways depending on the biogeographical area within Finland. In Finland, the Finnish Wildlife Agency grants hunting licences for moose, and when defining the amount of licences, a sustainable population of moose has to be ensured and damage caused by cervids has to be kept at a reasonable level.^36^ The agency consults with the relevant regional stakeholders over licences on an annual basis, and the amount of moose damage is one argument in these negotiations. Our modelling was aimed at developing a tool for moose management by which the effect of different moose population densities on the amount of damage can be estimated by MMAs.

## 2 MATERIALS AND METHODS

### 2.1 Moose population data

Moose population estimation in Finland is based on Bayesian population modelling, which synthesizes data from multiple sources (Appendix S1).^37^ The basis of the method is a population model where the population in a year produces the population of the subsequent year. The model is constructed to include the annual life cycle of moose: In spring new calves are born; in summer, wolves, bears and traffic cause mortality in the population; in autumn, hunting, wolves and traffic cause mortality; in winter, wolves and traffic cause mortality; and during the next spring, the surviving adult females produce new calves. At each phase of the cycle, the number and fates of adult males and females and juvenile males and females are recorded separately. The moose numbers are based on the estimates of moose population after hunting. Recruitment and sex ratio are inferred from the number of observed moose in the different categories and are obtained from moose observation cards completed by practically all moose hunting clubs. Mortality due to large carnivores is based on estimates of large carnivore populations, and mortality due to moose–vehicle collisions on statistics from the Finnish Transport and Communications Agency.^38^ Mortality due to hunting is based on the official hunting bag of the Finnish Wildlife Agency. The model run starts from several years before the year for which population estimates are calculated, and subsequent populations must be biologically compatible – that is, the population in each year must have been able to produce at least the bag hunted in the subsequent year. Distribution for the moose abundances in the analyzed time series are computed such that the fit between the modelled numbers and dynamics obtained from observation per unit effort (obtained from moose observation cards), moose population estimated by hunters, the number of moose–vehicle collisions and, in some areas, from aerial surveys is optimal.^39^

**Figure 1:**
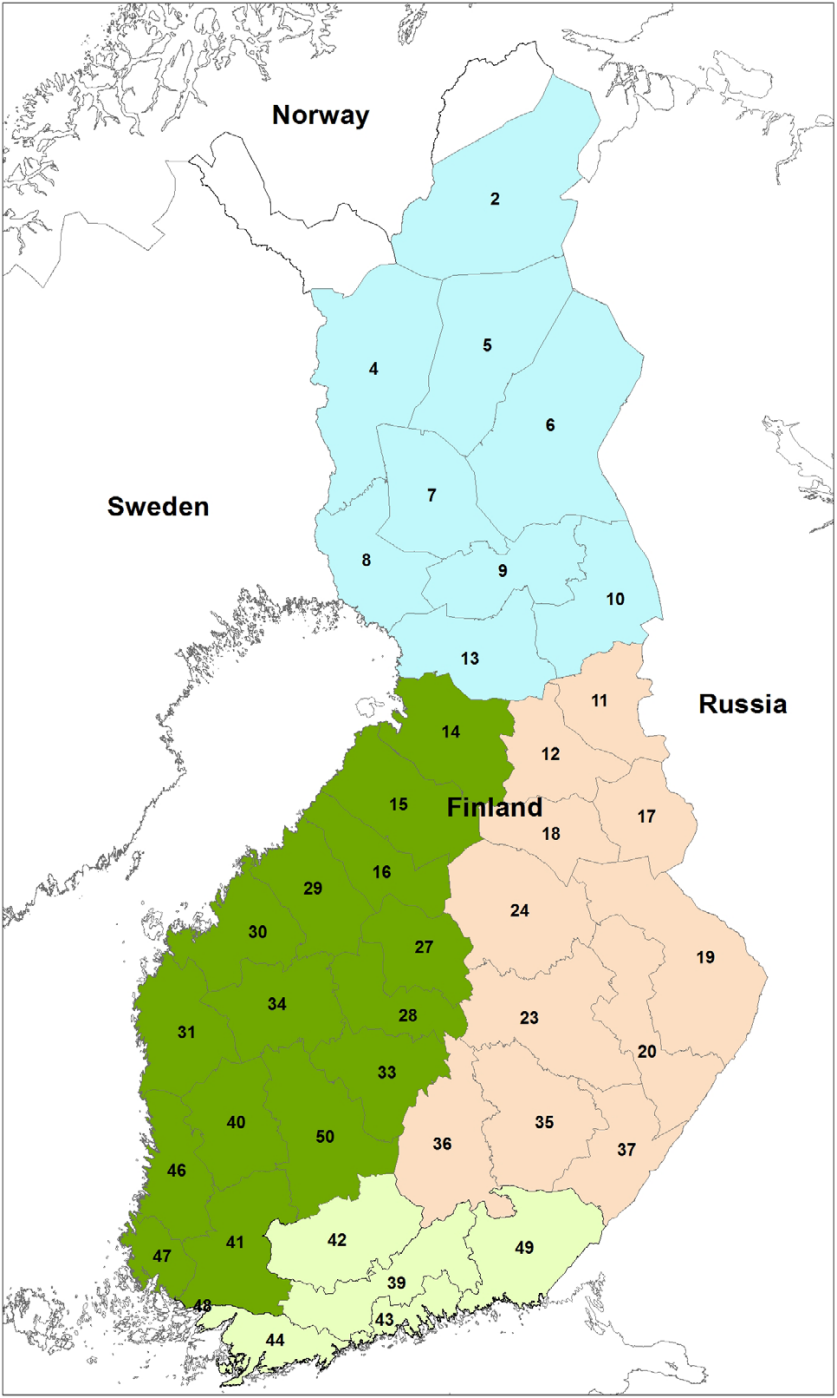
Moose management areas (MMA) used in the analysis. The smallest MMAs were merged to fulfill a minimum area of 500 000 ha. Each MMA was further associated to one of four zones representing different biogeographical conditions in Finland (blue, Lapland; orange, Eastern Finland; light green, Southern Finland; dark green, Western Finland).

Finland is divided into 59 MMAs for moose management purposes, and the average size of an MMA is 5112 km^2^ (SD 3327 km^2^). The annual estimated moose population sizes for modelling were calculated for each MMA, and the years where moose damage estimates (see Section 2.2) were available. We merged some adjacent moose management areas to get a minimum size of 5000 km^2^ land area for each study unit to guarantee an adequate number of NFI field plots for each MMA and more evenly distributed areas among MMAs. The catch‐all study units ‘MMAs’ can include both the original MMAs and merged MMAs. The two most northernmost MMAs were excluded from the study because the forest area comprises only 12% and 4.6% (respectively) of the land area. A total of 41 MMAs was used in modelling (Fig. 1). Moose population density estimates for each MMA were calculated by dividing population estimates by total land area, forest area or by the area of seedling stands with different dominant species.

Each MMA was further associated with one of four zones (Fig. 1) that we roughly delineated according to biogeographical regions of Finland.^40^ The Eastern Finland Zone covers the Finnish Lakeland, Northern Carelia and Kainuu regions, which belong to hemiboreal and middle boreal vegetation zones. The Lapland Zone belongs mostly to the northern boreal vegetation zone, consisting of the Kainuu–Kuusamo areas, North Ostrobothnia and Forest Lapland; the most southwestern part belongs to the middle boreal vegetation zone. The Southern Finland Zone covers mostly the southern boreal vegetation zone, with hemiboreal vegetation zones in the most southwestern part of Finland. The Western Finland Zone belongs mostly to the middle boreal vegetation zone, and the most southern parts of it to the south boreal vegetation zone.

### 2.2 Forest resource and moose damage data

We used the field measurements of the 10th (2004–2008) and 11th (2009–2013) NFIs.^9^, ^41^, ^42^ Systematic cluster sampling was used in both NFIs, and the average distance between neighbouring clusters varied from 6 km in South Finland to 10 km in North Finland. In NFI10, the number of sample plots per cluster varied from 12 (9 in NFI11) in South Finland to 14 (12 also in NFI11 permanent clusters) in Central Finland. In the NFI, stands were defined as units that are homogeneous in respect of site, growing stock and recommended future management. The stand description included >100 variables, including site, growing stock, damage, main tree species and the development class (successive phase) of forest (Table S1). Using the entire five‐year data for both NFIs separately, we calculated the area estimates for total land area, total forestry land (tree growth >1 m^3^ ha^−1^), development classes of stands by dominant tree species and moose damage for each MMA. These area estimates were based on the number of NFI field plots in the domain in question, the total number of NFI field plots in the MMA and the total land area of the MMA.^9^, ^41^

Damage was assessed at stand level, and the following variables were recorded: damage agent, symptoms of damage, severity of damage (mild, intermediate, severe, total) and time (Table S2). At maximum, the two most severe damage agents per stand and tree species were registered, and the severity of the damage was the sum effect of all the damage agents in the stand. In this study, the areas of intermediate, severe and total moose damage were used if the damage was classified as continuous (Table S2). These three damage classes reflected a significantly decreased quality of seedling stands. Continuous damage means that fresh browsing was still visible at the time of the inventory. In NFI10, 76% of damage cases were in advanced seedling stands, indicating that they were almost certainly caused by moose.^11^ In Southern Finland, some damage in young seedling stands was probably caused by white‐tailed deer and roe deer, but there were no inventory results available.

### 2.3 Statistical analysis and modelling

The total area of continuous moose damage (km^2^) in seedling stands per MMA and per both NFI inventory periods separately was used as a dependent variable in the modelling. Modelling was based on the theoretical framework of moose‐cover‐browsing material availability, and several combinations of the explanatory variables (Table 1) were tested during modelling.

**Table 1:** Variables that were tested during modelling and their mean, SD, median, minimum and maximum values

Variable	Mean	S.D.	Median	Min.	Max.
Total land area (km^2^)	7053	3.57	6710	1013	17 510
Total forest area (site productivity ≥1 m^3^ ha^−1^ yr^−1^), (km^2^)	4864	2012	4655	383	11 999
Proportion of forestry land area (of land area) (%)	55	15	59	7	76
Area of Scots pine seedling stands (km^2^)	535	337	450	31	1943
Area of Norway spruce seedling stands (km^2^)	297	344	214	0	1037
Area of deciduous seedling stands (km^2^)	73	51	64	8	213
Area of seedling stands (km^2^)	905	396	862	47	2250
Area of clear‐cuttings (km^2^)	66	33	61	0	164
Area of mature stands (km^2^)	2126	857	2004	241	4818
Proportion of Scots pine seedling stands of forest area (%)	11	4	10	4	20
Proportion of Norway spruce seedling stands of forest area(%)	10	2	10	5	12
Proportion of deciduous seedling stands of forest area (%)	2	1	1	0	6
Proportion of all seedling stands of forest area (%)	22	5	21	14	40
Moose density 10 km^−2^ land area	4	1	4	1	8
Moose density 10 km^−2^ Scots pine seedling stands	61	35	53	10	195
Proportion of mature stands (%)	45	10	45	27	72
Zone (four categories)	Lapland: *n* = 18 (22%), Western Finland = 30 (36%), Eastern Finland = 22 (27%), Southern Finland = 12 (15%)				

See SuppInfo 2.docx for the definition of variables.

In order to control for the varying sizes of MMAs, the land area, total forestry land area or proportion of forestry land area per MMA were used as base covariates in models. Forestry land area consisted of different forest development classes (forest succession phases) having different importance to moose: seedling stands serve as major food resources, and mature stands as shelter and an easy‐access living environment in snowy conditions.^43^ Most of the moose damage occurs in Scots pine‐dominated seedling stands and, therefore, we also tested the proportions of seedling stands with different dominant tree species.^11^ Differences among biogeographical zones (Lapland, and Western, Eastern and Southern Finland) and the interactions of the explanatory variables also were tested in candidate models. Owing to the high number of variables, it was not feasible to test all combinations of the variables and, therefore, we tested only those combinations that described the most probable food‐cover variables for moose.^15^, ^44^ Our modelling aimed at good forecast performance and, therefore, we adopted an approach that regards model construction as a process of testing several options rather than building the most parsimonious model.^45^

The linear mixed effects models used can be written as:

(1) $$\log\left(y_{\mathrm{ij}}\right)=\beta_{0}+\sum_{k=1}^{l}\beta_{\mathrm{ki}}x_{\mathrm{ki}}+\mu_{0j}+\varepsilon_{\mathrm{ij}}$$

where *β*_0_ is a fixed intercept, *β*_*ki*_ are fixed coefficients that have been measured at MMA level (i), *x*_*ki*_ are fixed explanatory variables that have been measured at MMA level (i), *μ*_*0j*_ is a random NFI effect (random intercept at level j), *ε*_*ij*_ is residual (exponential spatial autocorrelation was assumed) and *k* is the number of fixed variables representing the MMA effects.

MMAs close to each other may be more similar to each other than to those further away, and this was considered in the model by using an exponential spatial correlation structure for the residuals. Because we used data from NFI10 and NFI11, the NFI number was considered as a random factor in the mixed‐effects model.

The model and its parameters, and the coefficient of determination were expressed in log‐transformed scale, but the predictions were back‐transformed using exponential transformation. The bias in the back‐transformation was corrected by multiplying the predicted values by the ratio of observed and predicted means.^46^ Model predictions for each variable were calculated using only the observed min–max values in the data and using median values for co‐variates.

The mixed models were constructed using the R package nlme. The coefficients of determination (*R* ^2^) for the mixed effects models were computed using R/mumin.^47^ All of the analyses were computed in the R statistical environment.^48^

## 3 RESULTS

**Figure 2:**
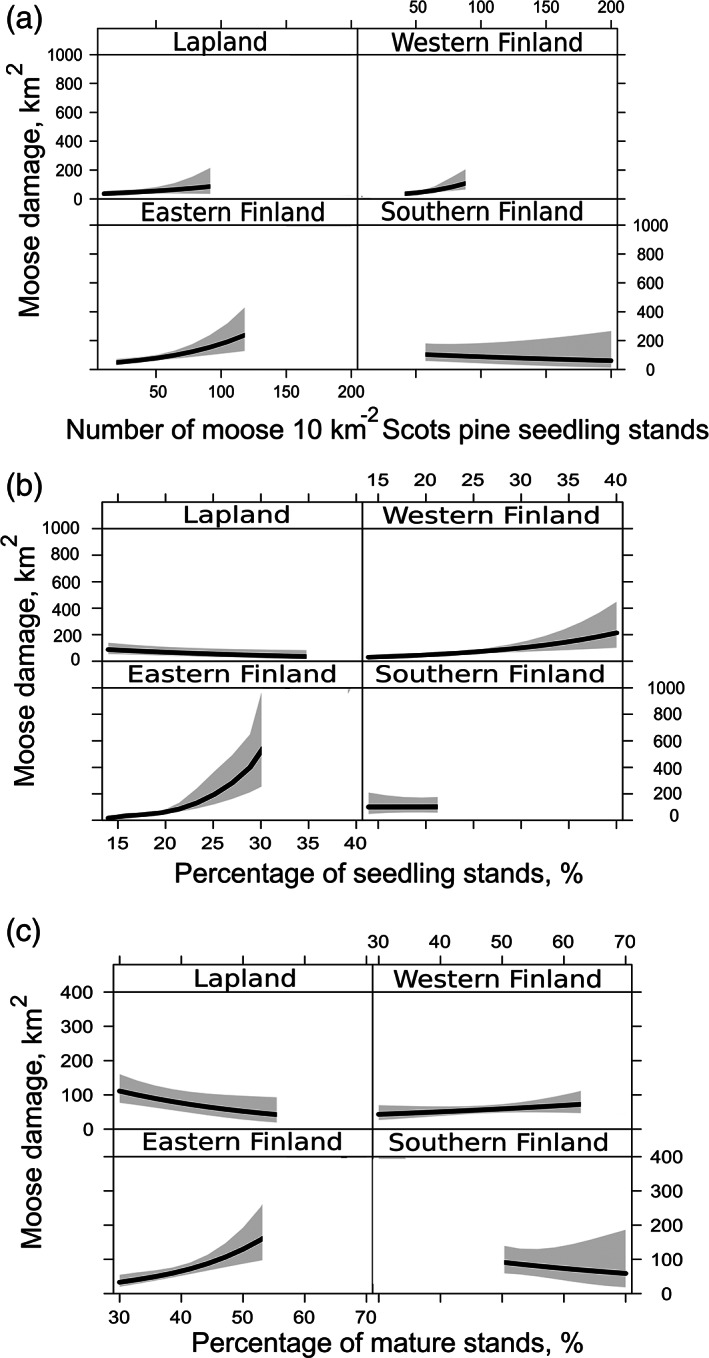
The predictions for moose *Alces alces* damage area in seedling stands (km^2^) and 95% confidence intervals for the main effects of (a) the number of moose 1000 ha^−1^. Scots pine seedling stands, (b) the proportion of all seedling stands and (c) the proportion of mature forests. Model includes interactions of Zone and all used variables.

**Figure 3:**
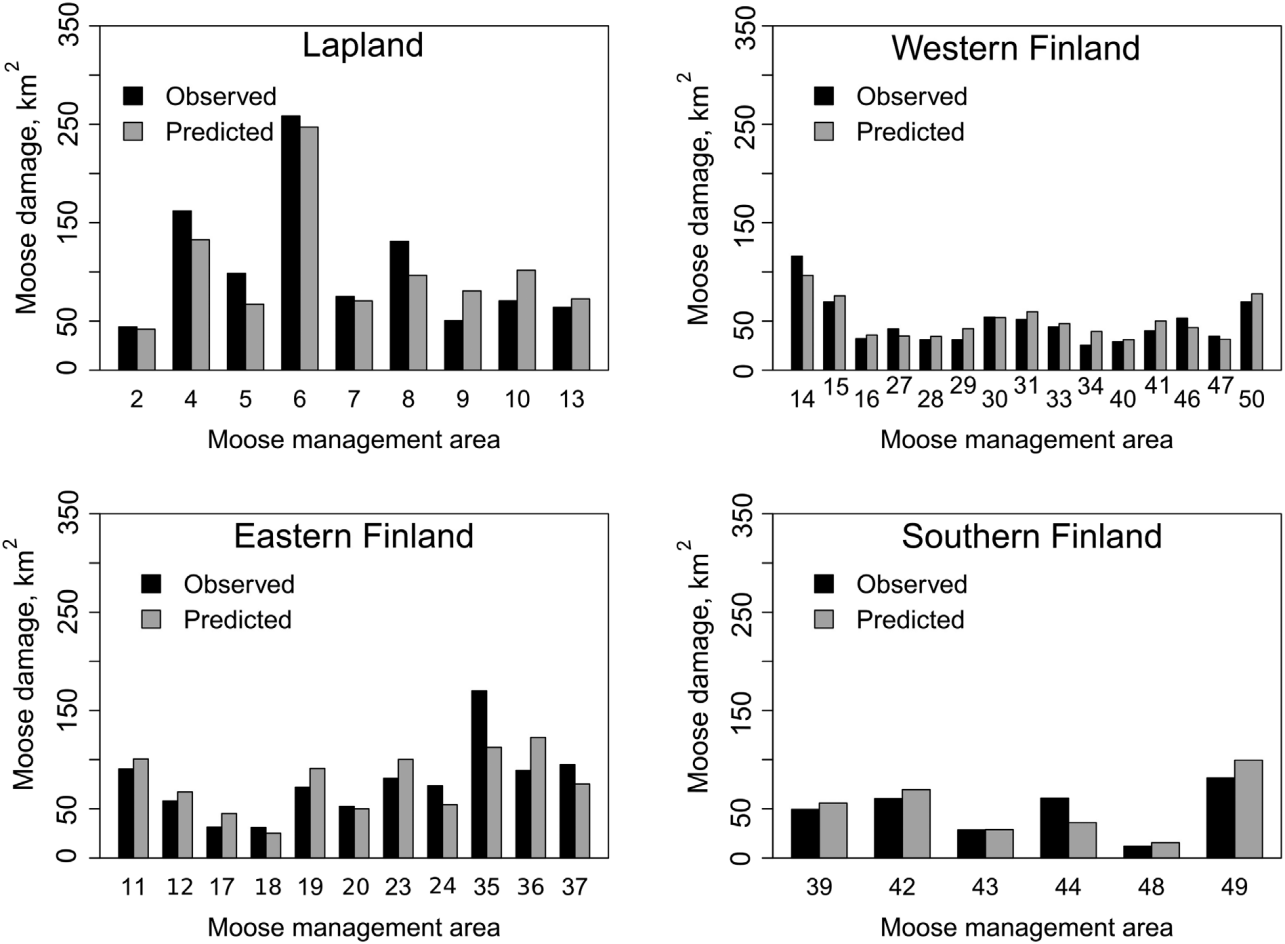
Observed *versus* predicted moose *Alces alces* damage in moose management areas by zones.

For the 23 models tested, the pseudo *R* ^2^ varied between 24% and 71.9%. Out of the best six models (Appendix S2) with pseudo *R* ^2^ > 60%, five included zone, total forest area, the proportions of seedling stands and mature forests as explanatory variables. The number of moose 1000 ha^−1^ land area or per Scots pine‐dominated seedling stand were the variables that best explained browsing pressure. The best model according to pseudo *R* ^2^ included total forest area, the proportion of Norway spruce [*Picea abies* (L.) Karsten] seedling stands and mature forests, and the number of moose per land area. The second‐best model had a pseudo *R* ^2^ of 69.5%, and it included total forest area, the proportion of seedling stands and mature forests, and the number of moose per Scots pine seedling stand. Because the majority of the moose damage occurred in non‐spruce‐dominated plantations, we found it feasible to present the model results for model 18 (Figs 2 and 3; Table 2; Appendix S2; see ‘Discussion' for other models’).

**Table 2:** The parameter estimates and tests of general linear mixed model for the continuous moose *Alces alces* damage

Variable fixed effects	Coefficient	SE	df	*t*/χ^2^	*P*
Intercept	5.055	1.213	64	4.167	<0.001
Zone (*ref. Lapland*)	—	—	3	39.465	<0.001
Western Finland	−5.974	1.213	64	−3.674	0.001
Eastern Finland	−10.406	1.884	64	−5.521	<0.001
Southern Finland	−0.321	1.950	64	−0.166	0.869
Forest area in MMA (km^2^)	2.24∙10^−4^	0.3∙10^−4^	64	7.539	<0.001
Moose density (animals 1000 ha^−1^ of Scots pine seedling stands)	0.011	0.005	64	1.444	0.154
Proportion of seedling stands (%)	−0.043	0.020	64	−2.207	0.031
Proportion of mature stands (%)	−0.038	0.017	64	−2.276	0.026
Zone * Moose density (animals 1000 ha^−1^ of Scots pine seedling stands)	—	—	3	15.782	0.001
Western Finland	0.012	0.010	64	1.375	0.174
Eastern Finland	5.82∙10^−3^	8.34∙10^−3^	64	0.698	0.490
Southern Finland	−0.014	0.008	64	−1.706	0.093
Zone * Proportion of seedling stands (*ref. Lapland*)	—	—	3	44.725	<0.001
Western Finland	0.118	0.027	64	4.319	<0.001
Eastern Finland	0.259	0.042	64	6.201	<0.001
Southern Finland	0.044	0.049	64	0.908	0.367
Zone * Proportion of mature stands (*ref. Lapland*)	—	—	3	18.251	<0.001
Western Finland	0.054	0.021	64	2.599	0.017
Eastern Finland	0.106	0.026	64	4.115	0.002
Southern Finland	0.016	0.034	64	0.469	0.641
**Random effects (variances) and range of exponential correlation**	**Variance**	**Range**			
Exponential spatial correlation		0.046			
NFI effect	5.620e10^−10^				
Residual	0.140				

SE, standard error, *t* and χ^2^ values, test values for the parameter estimates or type III ANOVA (deviance) tests; df, degrees of freedom; MMA, Moose Management Area. *R* ^2^ for marginal model was 69.4% (the variation explained by the fixed predictors).

The area of moose‐damaged stands increased along with increasing moose density per Scots pine seedling stand [Fig. 2(a)] in all zones except Southern Finland, where the trend was slightly decreasing. However, the 95% confidence interval of the predictions also was the largest in Southern Finland for the whole range of the moose population density, which makes the predictions less certain than in other zones. Probably as a consequence of the small number of observations, the confidence interval broadened towards the largest values in all zones.

The predicted area of moose damage per proportion of seedling stands increased in all zones except Lapland, where the trend was descending [Fig. 2(b)]. An increasing proportion of mature forests increased the predicted amount of damage in Western and Southern Finland, whereas the outcome was the opposite for Lapland and Southern Finland [Fig. 2(c)]. For all models, the performance was better when the interaction terms Zone × Var_n_ were added to the models than when using only the fixed effects of variables. The comparison of model predictions and observed values of the area of moose damage in each MMA showed a good match in all zones (Fig. 3).

## 4 DISCUSSION

In general, the area of moose damage in seedling stands increased with the increasing proportion of seedling stands and moose population density [but see Fig. 2(c) for Lapland]. The results support our hypothesis and are in line with previous studies, which have found that young forests contain the highest amount of browse species for moose and that the relative consumption is highest in young forests.^17^ However, the proportion of mature forests also was a significant variable in five of the six best models. Older forests also contain quite a large amount of browsing material, and they are an important factor explaining variation in consumption and damage.^17^, ^43^ A mosaic of seedling stands and mature forests explains the browsing and habitat use of moose.^15^, ^43^, ^44^, ^49^ In snowy environments, mature forests enable moose to move with a lower energy cost, and owing to the relatively small stand size, the distance between cover and food resources is short. In Southern Finland, however, the amount of mature forests might be linked simply to the amount of forest area as the landscape is fragmented by inhabited areas and agricultural land.

The best six models included a significant interaction term, Zone, which we originally included to account for the biogeographical variation in Finland. This is in line with previous studies, which have found several factors such as climate and snow, bedrock and soil, forage coverage, habitat patterns, inhabited areas, period of growth as well as competition with other deer species as plausible factors that explain regional variation in moose habitat selection and moose damage.^16^, ^19^, ^33^, ^34^, ^43^, ^50^, ^51^ As the interaction with Zone was significant also with the proportions of plantations and mature forests, our results indicate that there is regional variation in factors that are directly related to the moose forage availability and, consequently, the amount of damage. Owing to the limited amount of MMAs, we had to make the Zone division rather coarse to have enough observations per Zone. However, our results show that even this kind of zoning clearly improves the performance of the model, and it should be included in models as a proxy if direct measures of Zone‐related variables are not available. The significant effect of Zone also implies that moose management should be adjusted to local conditions for better moose damage control.^31^, ^32^

Judged with pseudo *R* ^2^, spruce‐dominated seedling stands together with the proportion of mature stands and the number of moose per land area explained the area of damaged plantations best. This was an unexpected result, as moose use mainly Scots pine in winter, and spruce is only seldom consumed in Fennoscandia.^23^, ^52^ An intuitive explanation is that when the amount of spruce‐dominated seedling stands is high, the amount of other tree species seedling stands is inevitably less, which increases browsing pressure in these. Another explanation could be that the proportion of spruce seedling stands serves as a proxy for more fertile soils, and thus for better food quality and quantities. This would be in line with the finding that there were more moose‐damaged Scots pine plantations in areas with higher amounts of nutrient‐rich bedrocks and soils.^16^ However, our results contrast with those from previous studies that have found the intensity of browsing on Scots pine to follow the variation in the abundance of other browsing species.^32^, ^34^ At the scale similar to our MMAs, pine browsing has been found to be negatively associated with the availability and quality of alternative browsing species.^32^, ^34^ The different results might be partly explained by how browsing is measured in different studies. In our modelling, browsing in seedling stands had to be continuous and the degree of damage at least intermediate. This probably covers only part of the total amount of browsing and habitats used by moose, as up to ≈42% of the total consumption has been measured to occur in older forests in some studies.^17^

Our results support the idea that moose management should be applied at scales that are large enough to cover the variation in local moose populations and the amount and quality of browsing species, as well as other factors influencing the habitat selection of moose.^32^, ^34^, ^43^, ^53^ Our models provide a tool for assessing the effect of different moose densities on the amount of damage as they can, in principle, be assessed for each MMA. NFI‐based forest and moose damage variables can be regarded as reliable at the level of MMA, as the number of sample plots is statistically determined.^41^ The accuracy of the estimated number of moose cannot be statistically calculated in a similar way as NFI estimates, and the migration of moose between summer and winter pastures might, to some degree, change the amount of moose per adjacent MMAs between hunting season and winter.^51^ However, because there was a statistically significant dependence between moose density and damage, moose population estimates can be regarded as reliable enough to capture the variation among MMAs.

The density of moose per area of Scots pine seedling stands and the number of moose per land area were significant factors in three of the six best models. Thus, our results do not give clear support to the idea that the amount of moose should be determined by using more detailed habitats than land area only. However, the models included the proportion of all seedling stands or Scots pine dominated seedling stands as a covariate and are thus included in the assessment of moose damage by local main food resources.

Finally, our results show that this modelling scheme can be used as a tool for assessing more detailed moose population – moose damage dependency for regional decision‐making. Although the time span for collecting NFI data for the whole country is five years, the change in forest landscapes resulting from logging and forest growth is probably not detrimental for the usability of the model.

## 5 CONCLUSION

The results indicate that the association between the amount of moose damage and forest resources can be modelled with adequate accuracy. Models can be used in analyzing the regional effects of moose population density and the amount of food resources on the amount of moose damage. This information can be used in reconciling sustainable moose population levels and the amount of damage in moose management decision‐making.

## CONFLICT OF INTEREST

The authors state that there are no conflicts of interest with any parties.

## Supporting information


**Appendix S1.** Supporting Information

**Appendix S2.** Supporting Information

**Table S1.** Nikula, A., Matala, J., Hallikainen, V., Pusenius, J., Ihalainen, A., Kukko, T. and Korhonen, K.T. Modelling the effect of moose Alces alces population density and regional forest structure on the amount of damage in forest seedling stands. Pest Management Science.

**Table S2.** Nikula, A., Matala, J., Hallikainen, V., Pusenius, J., Ihalainen, A., Kukko, T. and Korhonen, K.T. Modelling the effect of moose Alces alces population density and regional forest structure on the amount of damage in forest seedling stands. Pest Management Science.
