# Supplementary material for: Modelling the effect of moose Alces alces population density and regional forest structure on the amount of damage in forest seedling stands
Source: Pest Manag Sci. 2020 Sep 28;77(2):620–7. doi: 10.1002/ps.6081 (PMC7821013; doi:10.1002/ps.6081)
Supplement: Supplementary file 1 — Appendix S1. Supporting Information [file PS-77-620-s001.docx]

**Appendix S1**

Nikula, A., Matala, J., Hallikainen, V., Pusenius, J., Ihalainen, A., Kukko, T. and Korhonen, K.T. Modelling the effect of moose *Alces alces* population density and regional forest structure on the amount of damage in forest seedling stands. *Pest Management Science*. https://doi.org/10.1002/ps.6081.

**On the estimation of moose density**

The abundance of moose within 60 moose management areas (MMA) was estimated with a Bayesian state-space model. In the estimation, we utilized the generalized approach presented by Buckland et al.^1^ The grand model was built by: a) prior distributions for the initial states of moose population; b) prior distributions for model parameters; c) models for population processes; and d) observation models (likelihoods) for the multiple sources of data.

The core of the method was a temporal population dynamics model in which the annual processes of age increments, reproduction, hunting, predation, traffic kills and other mortality causes were modelled as consecutive pulses. The four age–sex groups modelled were: adult males (bulls), adult females (cows), male calves and female calves. Multiple sources of data were linked to the population at appropriate phases of the moose reproductive cycle.

Age increments were constructed in a deterministic process keeping adult moose unchanged and shifting calves into corresponding adult sub-populations. Reproduction was modelled by a multinomial process: each cow may produce zero, one, or two calves with probabilities estimated by moose observations.^2^ Hunting was a well-known process, as all hunters report their harvest via a tailored online tool. Predation of large carnivores was evaluated based on the areal estimates of brown bear and wolf populations and previously published predation rates.^3-6^ Traffic kills of moose were recorded by the Finnish Transport Infrastructure Agency, and decreased the population in the model. Finally, we allowed natural mortality to reduce the population by an unknown constant proportion.

The main source of data supporting the population model was moose observations gathered from the moose observation cards and, from 2016 onwards, the online moose information system ‘Oma riista’ hosted by The Finnish Wildlife Agency.^7,8^ Moose observations were assumed to reflect the daily state of local moose population, and observed changes in the ratios of sub-populations during the hunting season were modelled within the state-space model by an integrated sequential change-in-ratio method.^9^ Another important source of data was the aerial counts. We have accessed line transect counts performed by the game management units and aerial counts conducted in some northern MMAs by a distance sampling protocol.^10^

The moose populations were modelled for a period of 20 years (2000–2019) including several annual population processes and multiple annual observation models. The analytic solution was unfeasible, so the population abundance was calculated using a Markov chain Monte Carlo simulation in the software applications R and JAGS (R Core Team 2017, Plummer 2012).^11,12^ The numerical solution gained by simulation was an empirical sample of a very high-dimensional posterior distribution. In practice, by ignoring other dimensions of the posterior, we had at hand a collection of probable moose population trajectories for the study period for each MMA.

Medians of the annual marginal posteriors of the moose densities were estimated. Moose population estimates derived in such a way were interpreted to be close to the most probable moose population trajectory that has produced all the observed data, given the assumptions of moose population dynamics. Finally, the estimates of moose abundance were translated to moose densities by dividing them by the land area of corresponding MMAs.

References

1 Buckland, S. T., Anderson, D. R., Burnham, K. P., & Laake, J. L. Distance sampling. *Encyclopedia of Biostatistics* 2. (2005)

2 Nygrén, T. The potential for multiple fecundity of moose in Finland. *Alces* **39**:89-107. (2003)

3 Heikkinen, S., Kojola, I., & Mäntyniemi, S. Karhukanta Suomessa 2018. Luonnonvara- ja biotalouden tutkimus 16/2019. Luonnonvarakeskus. Helsinki. 17 s. (2019)

4 Heikkinen, S., Kojola, I., Mäntyniemi, S., Holmala, K., & Härkälä, A. Susikanta Suomessa maaliskuussa 2020. Luonnonvara- ja biotalouden tutkimus 35/2019, 92 p. (2020)

5 Swenson, J. E., Dahle, B., Busk, H., Opseth, O. L. E., Johansen, T., Söderberg, A., ... & Cederlund, G.. Predation on moose calves by European brown bears. *J Wildlife Manage* **71**:1993-1997. (2007)

6 Hayes, R. D., & Harestad, A. S. Wolf functional response and regulation of moose in the Yukon. *Can J Zool* **78**:60-66. (2000)

7 Nygrén, T., & Pesonen, M.. The moose population (Alces alces L.) and methods of moose management in Finland, 1975–89. *Finnish Game Research* **48**:46-53. (1993)

8 Kukko, T.. Oma riista: tutkimuksen apuna (in finnish). Hyvinvoiva riista - kestävä riistatalous, riistapäivät 2017, 18.–19.1. 2017, hotelli Scandic, Kuopio: Abstracts. (2017)

9 Skalski, J. R., & Millspaugh, J. J.. Application of multidimensional change‐in‐ratio methods using program USER. *Wildl Soc Bull* **34**:433-439. (2006)

10 Buckland, S. T., Newman, K. B., Thomas, L., & Koesters, N. B.. State-space models for the dynamics of wild animal populations. *Ecol Model* 171(1-2), 157-175. (2004)

11 R Core Team. R: A language and environment for statistical computing. R Foundation for Statistical Computing, Vienna, Austria. URL https://www.R-project.org/ [accessed August 6th 2020].

12 Plummer, M. JAGS Version 3.3. 0 User manual. International Agency for Research on Cancer, Lyon, France. (2012)
