# Supplementary material for: Modelling the effect of moose Alces alces population density and regional forest structure on the amount of damage in forest seedling stands
Source: Pest Manag Sci. 2020 Sep 28;77(2):620–7. doi: 10.1002/ps.6081 (PMC7821013; doi:10.1002/ps.6081)
Supplement: Supplementary file 2 — Appendix S2. Supporting Information [file PS-77-620-s002.docx]

**Appendix S2**

|  | **Model 12** |  |  | **Model 14** |  |  | **Model 18** |  |  |  | **Model 20** |  |  |  | **Model 22** |  |  |  | **Model 23** |  |  |
| --- | --- | --- | --- | --- | --- | --- | --- | --- | --- | --- | --- | --- | --- | --- | --- | --- | --- | --- | --- | --- | --- |
|  | Estimate | S.E. |  | Estimate | S.E.. |  | Estimate | | S.E.. |  | Estimate | | S.E.. |  | Estimate | | S.E.. |  | Estimate | | S.E.. |
| (Intercept) | 3.885*** | 0.797 |  | 2.114 ^n.s.^ | 1.406 |  | 5.055*** | | 1.213 |  | 4.338*** | | 0.878 |  | 6.040*** | | 0.953 |  | 5.157*** | | 0.970 |
| Zone (ref. Lapland) |  |  |  |  |  |  |  | |  |  |  | |  |  |  | |  |  |  | |  |
| -Western Finland | -2.532** | 0.846 |  | -4.252** | 1.277 |  | -5.975*** | | 1.626 |  | -4.025*** | | 1.102 |  | -5.780*** | | 1.273 |  | -4.631** | | 1.308 |
| -Eastern Finland | -4.259*** | 1.115 |  | -7.859*** | 1.738 |  | -10.406*** | | 1.885 |  | -7.534*** | | 1.28 |  | -9.668*** | | 1.685 |  | -9.722*** | | 1.730 |
| -Southern Finland | 3.667* | 1.422 |  | 1.184 ^n.s.^ | 1.949 |  | -0.320 ^n.s.^ | | 1.930 |  | 1.325 ^n.s.^ | | 2.076 |  | -0.584 ^n.s.^ | | 1.906 |  | 0.209 ^n.s.^ | | 1.940 |
| Land area, km^2^ |  |  |  |  |  |  |  | |  |  |  | |  |  | 0.0002*** | | 2.19E-05 |  |  | |  |
| Forest area MMA, km^2^ | 0.0002*** | 2.88E-05 |  | 0.0002*** | 0.00002 |  | 0.0002*** | | 2.97E-05 |  | 0.0002*** | | 2.68E-05 |  |  | |  |  | 0.0002*** | | 3.16E-05 |
| Moose density 10 km^-2^ Scots pine seedling stands | -0.016^n.s.^ | 0.015 |  |  |  |  | 0.010 ^n.s.^ | | 0.007 |  |  | |  |  |  | |  |  | 0.010** | | 0.003 |
| Moose density 10 km^-2^ land area |  |  |  | 0.192*** | 0.054 |  |  | |  |  | 0.229*** | | 0.047 |  | 0.245*** | | 0.051 |  |  | |  |
| Proportion of seedling stands, % | -0.064* | 0.027 |  | 0.041 ^n.s.^ | 0.049 |  | -0.043* | | 0.019 |  |  | |  |  | -0.084*** | | 0.019 |  | -0.047* | | 0.019 |
| Proportion of spruce seedling stands, % |  |  |  |  |  |  |  | |  |  | -0.131*** | | 0.036 |  |  | |  |  |  | |  |
| Proportion of mature stands, % |  |  |  | 0.037 ^n.s.^ | 0.030 |  | -0.038* | | 0.017 |  | -0.021 ^n.s.^ | | 0.015 |  | -0.062*** | | 0.016 |  | -0.040** | | 0.015 |
| Zone*Proportion of seedling stands, % |  |  |  |  |  |  |  | |  |  |  | |  |  |  | |  |  |  | |  |
| -Western Finland | 0.068** | 0.022 |  | 0.097*** | 0.025 |  | 0.118*** | | 0.027 |  |  | |  |  | 0.105*** | | 0.025 |  | 0.100** | | 0.027 |
| -Eastern Finland | 0.172*** | 0.040 |  | 0.193*** | 0.0375 |  | 0.259*** | | 0.042 |  |  | |  |  | 0.207*** | | 0.037 |  | 0.236*** | | 0.041 |
| -Southern Finland | -0.134* | 0.066 |  | 0.107* | 0.0479 |  | 0.044 ^n.s.^ | | 0.049 |  |  | |  |  | 0.089* | | 0.043 |  | 0.116* | | 0.047 |
| Zone*Moose per 10 km^2^ Scots pine seedling stands |  |  |  |  |  |  |  | |  |  |  | |  |  |  | |  |  |  | |  |
| -Western Finland | 0.013^n.s.^ | 0.010 |  |  |  |  | 0.014 ^n.s.^ | | 0.010 |  |  | |  |  |  | |  |  |  | |  |
| -Eastern Finland | 0.009n^n.s.^ | 0.009 |  |  |  |  | 0.006 ^n.s.^ | | 0.008 |  |  | |  |  |  | |  |  |  | |  |
| -Southern Finland | -0.017* | 0.008 |  |  |  |  | -0.014 ^n.s.^ | | 0.008 |  |  | |  |  |  | |  |  |  | |  |
| Moose density 10 km^-2^ Scots pine seedling stands*Proportion of seedling stands, % | 0.001** | 0.001 |  |  |  |  |  | |  |  |  | |  |  |  | |  |  |  | |  |
| Proportion of seedling stands*Proportion of mature stands |  |  |  | -0.003* | 0.001 |  |  | |  |  |  | |  |  |  | |  |  |  | |  |
| Zone*Proportion of mature stands, % |  |  |  |  |  |  |  | |  |  |  | |  |  | 0.082** | | 0.020 |  |  | |  |
| -Western Finland |  |  |  | 0.043* | 0.020 |  | 0.054* | | 0.021 |  | 0.039*** | | 0.017 |  | 0.135*** | | 0.026 |  | 0.051* | | 0.020 |
| -Eastern Finland |  |  |  | 0.088** | 0.0274 |  | 0.106*** | | 0.026 |  | 0.051* | | 0.020 |  | -0.005 ^n.s.^ | | 0.030 |  | 0.120** | | 0.026 |
| -Southern Finland |  |  |  | -0.058 ^n.s.^ | 0.0301 |  | 0.016 ^n.s.^ | | 0.034 |  | -0.048 ^n.s.^ | | 0.029 |  |  | |  |  | -0.044 ^n.s.^ | | 0.032 |
| Zone*Proportion of spruce seedling stands, % |  |  |  |  |  |  |  | |  |  |  | |  |  |  | |  |  |  | |  |
| -Western Finland |  |  |  |  |  |  |  | |  |  | 0.211*** | | 0.053 |  |  | |  |  |  | |  |
| -Eastern Finland |  |  |  |  |  |  |  | |  |  | 0.556*** | | 0.085 |  |  | |  |  |  | |  |
| -Southern Finland |  |  |  |  |  |  |  | |  |  | 0.169 ^n.s.^ | | 0.108 |  |  | |  |  |  | |  |
|  |  |  |  |  |  |  |  | |  |  |  | |  |  |  | |  |  |  | |  |
| Pseudo R^2^, % | 62.7 |  |  | 66.6 |  |  | 69.4 | |  |  | 71.9 | |  |  | 67.0 | |  |  | 63.3 | |  |

Six best models with Pseudo R^2^ >60% and their coefficient estimates (Estimate) and standard error of co-efficient estimate (S. E.). Significance for explanatory variables: *** = p<0.001, ** = p<0.01, * = p<0.05, not significant = n.s.

Nikula, A., Matala, J., Hallikainen, V., Pusenius, J., Ihalainen, A., Kukko, T. and Korhonen, K.T. Modelling the effect of moose *Alces alces* population density and regional forest structure on the amount of damage in forest seedling stands. *Pest Management Science*. https://doi.org/10.1002/ps.6081.
