# Supplementary material for: Modelling the effect of moose Alces alces population density and regional forest structure on the amount of damage in forest seedling stands
Source: Pest Manag Sci. 2020 Sep 28;77(2):620–7. doi: 10.1002/ps.6081 (PMC7821013; doi:10.1002/ps.6081)
Supplement: Supplementary file 3 — Table S1. Nikula, A., Matala, J., Hallikainen, V., Pusenius, J., Ihalainen, A., Kukko, T. and Korhonen, K.T. Modelling the effect of moose Alces alces population density and regional forest structure on the amount of damage in forest seedling stands. Pest Management Science. [file PS-77-620-s003.docx]

**Table S1**

|  | |  | |  | |
| --- | --- | --- | --- | --- | --- |
| **Variable** | | **Description** | | **Additional explanation** | |
| Total land area, km^2^ | | Includes all land-use classes of Finland. | |  | |
| Total forest area (site productivity  ≥1 m^3^ha^-1^y^-1^), km^2^ | | Forest area as stocked or temporarily unstocked land with potential capacity to produce a mean annual increment of at least 1 m^3^ of stem wood during the prescribed rotation under the most favourable stock conditions. Parks and yards were excluded. | |  | |
| Area of Scots pine seedling stands, km^2^ | Seedling stands where Scots pine was the most frequent species (by volume) in the dominant tree storey of the stand. | | Two seedling stand classes were combined : 1. Young seedling stand: a stand with a dominant height of the dominant tree species <1.3 m; 2. Advanced seedling stand: a stand with a dominant height of the dominant tree species >1.3 m. For a major part of the dominant trees the diameter at breast height was less than 8 cm, and for the largest trees less than 10 cm. | |  |
| Area of Norway spruce seedling stands, km^2^ | Norway spruce as dominant tree species. | |  |  |  |
| Area of deciduous seedling stands, km^2^ | Seedling stand where any of the deciduous species was dominant. Most often the dominant species is Silver birch (*Betula pendula* Roth). | |  |  |  |
| Area of seedling stands, km^2^ | | All seedling stands. | |  | |
| Area of clear-cuttings, km^2^ | | Temporarily unstocked regeneration stand: a treeless area with possible retention trees. Small groups of seedlings may also occur. | |  | |
| Area of mature stands, km^2^ | | Mature stand: a stand with a growing stock either old and/or large enough for the forest management goal. Regeneration cutting had not yet started. The maturity for regeneration was primarily determined with by the age of growing stock, and to some extent with by mean diameter. | |  | |

Table S1. Description and explanation of National Forest Inventory forest resource variables applied in this study.

Nikula, A., Matala, J., Hallikainen, V., Pusenius, J., Ihalainen, A., Kukko, T. and Korhonen, K.T. Modelling the effect of moose *Alces alces* population density and regional forest structure on the amount of damage in forest seedling stands. *Pest Management Science*. https://doi.org/10.1002/ps.6081.
