# Supplementary material for: Modelling the effect of moose Alces alces population density and regional forest structure on the amount of damage in forest seedling stands
Source: Pest Manag Sci. 2020 Sep 28;77(2):620–7. doi: 10.1002/ps.6081 (PMC7821013; doi:10.1002/ps.6081)
Supplement: Supplementary file 4 — Table S2. Nikula, A., Matala, J., Hallikainen, V., Pusenius, J., Ihalainen, A., Kukko, T. and Korhonen, K.T. Modelling the effect of moose Alces alces population density and regional forest structure on the amount of damage in forest seedling stands. Pest Management Science. [file PS-77-620-s004.docx]

| **Table S2** |  |  |
| --- | --- | --- |
| **Variable** | **Description** | **Additional explanation** |
|  |  |  |
| Damage symptoms | Visual assessment of the injury | In case of moose damage: broken or dry top (in the upper half of the crown), technical defects on stem, other crown malformations, defoliation. |
| Degree of damage | Apparent severity of damage at the stand level which was evaluated by change in silvicultural quality of the stand, see next variable row. Damage classes 1, 2 and 3 were utilized. | 0 mild damage, symptoms observed, but the damage does not reduce the silvicultural quality of the stand;  1 intermediate, the stand quality is reduced by one class;  2 severe, the stand quality is reduced by more than one class;  3 total, artificial regeneration is required. |
| Silvicultural quality of the stand | The division was based on species composition, dominant height, even spatial distribution, density and technical quality of the dominant tree storey. | 1 good  2 satisfactory  3 adequate  4 low-productive: the yield is so low that the stand should be regenerated before maturity. |
| Age of damage | The time when the damage has started and the possible continuing effect of the cause in the stand. In this study damage age classes 0, 1 and 3 were utilized which means that there was fresh damage at inventory year. | 0 started less than two years ago;  1 started 2–5 years ago and still continuing;  2 started 2–5 years ago, but ceased;  3 started more than 5 years ago and still continuing;  4 started more than 5 years ago but ceased. |

Table S2. Description and explanation of National Forest Inventory moose *Alces alces* damage variables applied in this study.

Nikula, A., Matala, J., Hallikainen, V., Pusenius, J., Ihalainen, A., Kukko, T. and Korhonen, K.T. Modelling the effect of moose *Alces alces* population density and regional forest structure on the amount of damage in forest seedling stands. *Pest Management Science*. https://doi.org/10.1002/ps.6081.
